# The Toxin-Antitoxin MazEF Drives Staphylococcus aureus Biofilm Formation, Antibiotic Tolerance, and Chronic Infection

**DOI:** 10.1128/mBio.01658-19

**Published:** 2019-11-26

**Authors:** Dongzhu Ma, Jonathan B. Mandell, Niles P. Donegan, Ambrose L. Cheung, Wanyan Ma, Scott Rothenberger, Robert M. Q. Shanks, Anthony R. Richardson, Kenneth L. Urish

**Affiliations:** a Arthritis and Arthroplasty Design Group, Department of Orthopaedic Surgery, College of Medicine, University of Pittsburgh, Pittsburgh, Pennsylvania, USA; b Department of Microbiology and Immunology, Geisel School of Medicine at Dartmouth, Hanover, New Hampshire, USA; c Clinical and Translational Science Institute, University of Pittsburgh, Pittsburgh, Pennsylvania, USA; d Department of Medicine, University of Pittsburgh, Pittsburgh, Pennsylvania, USA; e Department of Ophthalmology, University of Pittsburgh, Pittsburgh, Pennsylvania, USA; f Department of Microbiology and Molecular Genetics, University of Pittsburgh, Pittsburgh, Pennsylvania, USA; g The Bone and Joint Center, Magee-Womens Hospital of the University of Pittsburgh Medical Center, Pittsburgh, Pennsylvania, USA; h Department of Bioengineering, University of Pittsburgh, Pittsburgh, Pennsylvania, USA; i Department of Biomedical Engineering, Carnegie Mellon University, Pittsburgh, Pennsylvania, USA; University of Pittsburgh

**Keywords:** surgical infection, biofilm, MazF, *Staphylococcus aureus*, toxin-antitoxin (TA) systems, *icaADBC*, periprosthetic joint infection, surgical infection

## Abstract

Staphylococcus aureus is the major organism responsible for surgical implant infections. Antimicrobial treatment of these infections often fails, leading to expensive surgical intervention and increased risk of mortality to the patient. The challenge in treating these infections is associated with the high tolerance of S. aureus biofilm to antibiotics. MazEF, a toxin-antitoxin system, is thought to be an important regulator of this phenotype, but its physiological function in S. aureus is controversial. Here, we examined the role of MazEF in developing chronic infections by comparing growth and antibiotic tolerance phenotypes in three S. aureus strains to their corresponding strains with disruption of *mazF* expression. Strains lacking *mazF* production showed increased biofilm growth and decreased biofilm antibiotic tolerance. Deletion of *icaADBC* in the *mazF*::Tn background suppressed the growth phenotype observed with *mazF*-disrupted strains, suggesting the phenotype was *ica* dependent. We confirmed these phenotypes in our murine animal model. Loss of *mazF* resulted in increased bacterial burden and decreased survival rate of mice compared to its wild-type strain demonstrating that loss of the *mazF* gene caused an increase in S. aureus virulence. Although lack of *mazF* gene expression increased S. aureus virulence, it was more susceptible to antibiotics *in vivo*. Combined, the ability of *mazF* to inhibit biofilm formation and promote biofilm antibiotic tolerance plays a critical role in transitioning from an acute to chronic infection that is difficult to eradicate with antibiotics alone.

## Abstract (precis)

Surgical infections are one of the most common types of infections encountered in a hospital. Staphylococcus aureus is the most common pathogen associated with this infection. These infections are resilient and difficult to eradicate, as the bacteria form biofilm, a community of bacteria held together by an extracellular matrix. Compared to bacteria that are planktonic, bacteria in a biofilm are more resistant to antibiotics. The mechanism behind how bacteria develop this resistance and establish a chronic infection is unknown. We demonstrate that *mazEF*, a toxin-antitoxin gene, inhibits biofilm formation and promotes biofilm antibiotic tolerance which allows S. aureus to transition from an acute to chronic infection that cannot be eradicated with antibiotics but is less virulent. This gene not only makes the bacteria more tolerant to antibiotics but makes the bacteria more tolerant to the host.

## INTRODUCTION

Staphylococcus aureus is a Gram-positive pathogen associated with a variety of disease processes from self-limited abscesses to life-threatening sepsis. These episodes are typically acute and resolve over a limited time period to various degrees of morbidity and mortality (1). An exception is S. aureus-related surgical infection, especially those associated with medical devices. Surgical site infection is one of the most common health care-associated infections (2). Unlike the majority of S. aureus infections, these infections can be chronic, indolent, and challenging to treat.

Periprosthetic joint infection illustrates this challenge. Total knee arthroplasty is a common surgical procedure, and the most common reason for failure is infection, termed periprosthetic joint infection (3, 4). S. aureus periprosthetic joint infection can be culture negative for prolonged periods (5, 6), has high failure rates above 50% once treatment is initiated (5), and a 5-year mortality of 20% (7–9), higher than many common cancers (10). Similar to other surgical implant-associated infections, the challenge in treating this disease involves the ability of S. aureus to develop a chronic biofilm-associated infection tolerant to antibiotics (11, 12).

In Gram-positive bacteria, the mechanisms behind biofilm antibiotic tolerance and the ability to form chronic infections are poorly understood. It is suspected that toxin-antitoxin (TA) systems play an important role in these processes. Toxin-antitoxin systems encode a stable toxin protein capable of interfering with vital cellular processes and a labile antitoxin that counteracts the toxin (13–15). When a bacterial cell encounters a stress, i.e., antibiotics, the antitoxin is triggered to disassemble, and the toxin becomes activated to disrupt an essential bacterial metabolic process, inducing a state of dormancy. This is thought to render the bacteria tolerant to antibiotics, as there is no metabolic pathway to disrupt. TA systems are implicated in bacterial persisters and biofilm formation, induced through a decreased metabolic state (16, 17). Persisters are a subpopulation of bacteria that have a phenotypic tolerance to antibiotics (18, 19). In S. aureus, the most well-studied TA system is the MazEF module where MazF is a stable toxin that cleaves specific mRNA, and MazE is an unstable antitoxin that inhibits MazF (20). MazF is an endoribonuclease whose target is cleavage of single-stranded ACA sequences to inhibit translation (20–23). In Gram-negative and acid-fast species, TA system has been associated with antibiotic tolerance (24) and virulence (25). In S. aureus, the *mazEF* phenotype is controversial, and its physiological function in the disease process is unknown.

The objective of this study was to identify a phenotype associated with *mazEF* in the S. aureus disease process. We hypothesized that toxin-antitoxin systems like *mazEF* contribute to the ability to establish chronic infections and antibiotic-tolerant biofilms. Disruption of *mazF* expression in three different common S. aureus strains resulted in increased biofilm formation and loss of antibiotic tolerance compared to their wild-type strains on surgical implant material. In planktonic culture, when *mazF* disruption did alter growth, this was associated with antibiotic tolerance. In our animal model, the absence of *mazF* resulted in a more acute, pathogenic infection that was more susceptible to antibiotics. These phenotypes demonstrated that *mazF* expression resulted in lower growth and metabolic activity from decreased biofilm formation that allowed a transition from an acute to chronic biofilm infection and increased antibiotic tolerance.

## RESULTS

### Disruption of *mazF* is associated with increased biofilm formation on surgical implant material.

**FIG 1:**
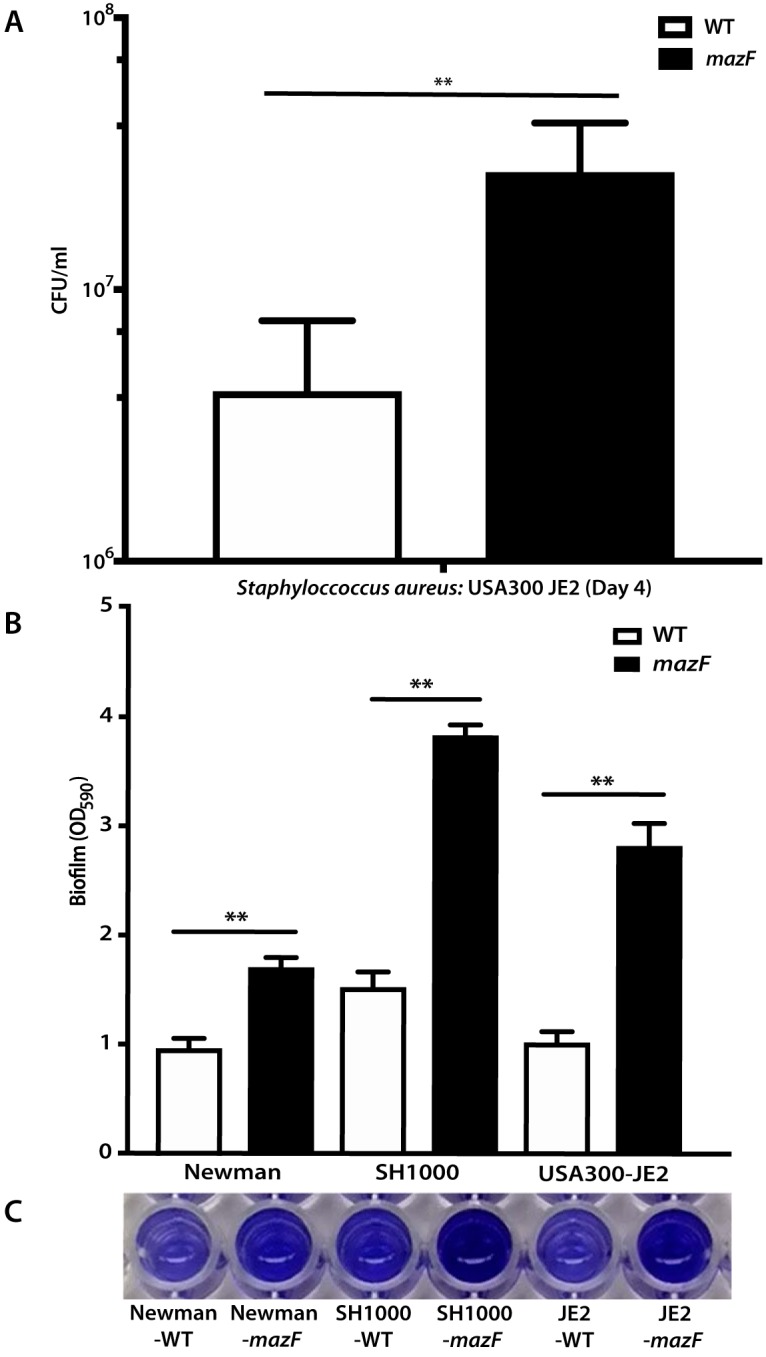
Loss of *mazF* expression increases biofilm formation in S. aureus. (A) S. aureus biofilm was cultured on surgical implant material (12-mm titanium rods) for 4 days to form mature biofilm, and biofilm growth was quantified by sonication and quantitative culture. (B) S. aureus strains were cultured on fibrinogen-coated 96-well polystyrene plates for 24 to 48 h. Biofilm formation was quantified by the crystal violet method, and the absorbance was measured at 590 nm. (C) Biofilm stained with crystal violet was dissolved in 30% acetic acid. All experiments were performed in triplicate. Values that are significantly different are indicated by a bar and asterisks as follows: **, *P* < 0.01. Error bars represent 95% confidence intervals (95% CI).

**FIG 2:**
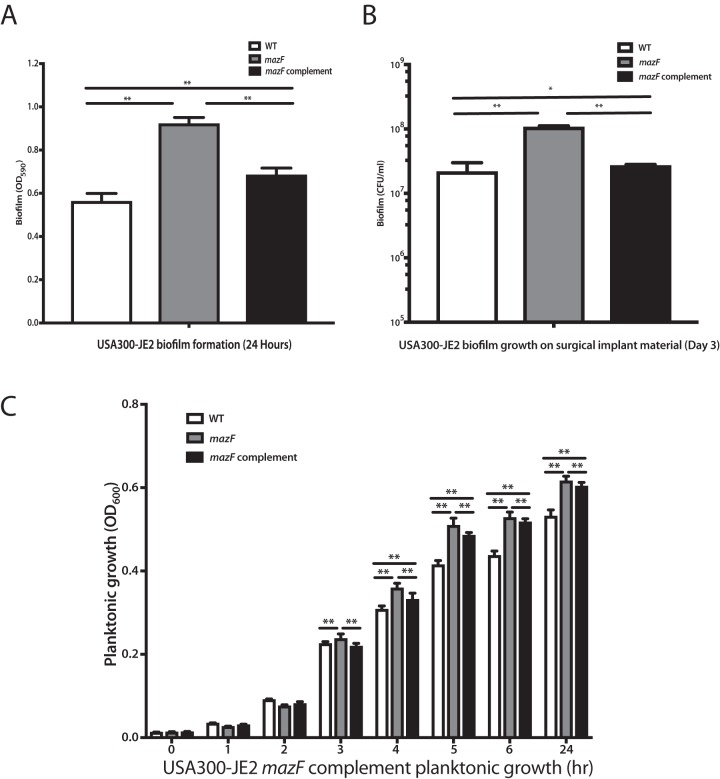
MazF complement reduces the planktonic cell growth and biofilm formation. A genomic complement approach was used to restore *mazF* expression in JE2 *mazF*::Tn, and the growth phenotype was reversed. Wild-type (WT) JE2 strain with an empty spectinomycin vector was used as a control. (A to C) Biofilm formation was measured by using the crystal violet assay (A) and titanium rod CFU assay (B), and planktonic growth measured using optical density (C) demonstrated that biofilm formation and planktonic growth was decreased in the *mazF* complemented strain. All experiments were performed in triplicate. Statistical significance: *, *P* < 0.05; **, *P* < 0.01. Error bars represent 95% CI (95% confidence intervals).

Toxin-antitoxin systems are associated with bacterial growth arrest (26–28). We hypothesized that the lack of *mazF* would result in increased biofilm formation from preventing growth inhibition. Mature S. aureus (USA300 JE2) biofilm was cultured on titanium rods, and quantitative culture was performed to assess biofilm mass. Disruption of *mazF* resulted in increased biofilm mass compared to parental strains (Fig. 1A). We observed similar results on two additional methicillin-sensitive S. aureus (MSSA) strains deleted for *mazF*, Newman (29) and SH1000 (30) (see Fig. S1 in the supplemental material). These experiments were repeated, and biofilm was cultured on polystyrene and quantified with crystal violet assay. A loss of *mazF* expression again resulted in increased biofilm mass on fibrinogen-coated wells in all three strains compared to the wild-type strains (Fig. 1B and C). To confirm the observed phenotype of *mazF* in S. aureus, we restored *mazF* expression in *trans* and observed a decrease in biofilm formation (Fig. 2).

10.1128/mBio.01658-19.1 FIG S1 Loss of *mazF* increases biofilm formation on surgical implant material in S. aureus. Biofilm was cultured on surgical implant material (titanium rods [12 mm]) for 4 days to form mature biofilm, and the biofilm growth was quantified by sonication, plating, and enumeration for JE2, Newman, and SH1000 strains, respectively. All experiments were performed in triplicate. **, *P* < 0.01. Error bars represent 95% CI (95% confidence intervals). Download FIG S1, EPS file, 0.8 MB. Copyright © 2019 Ma et al. 2019 Ma et al. This content is distributed under the terms of the Creative Commons Attribution 4.0 International license.

### Loss of *mazF* expression decreases biofilm antibiotic tolerance.

**FIG 3:**
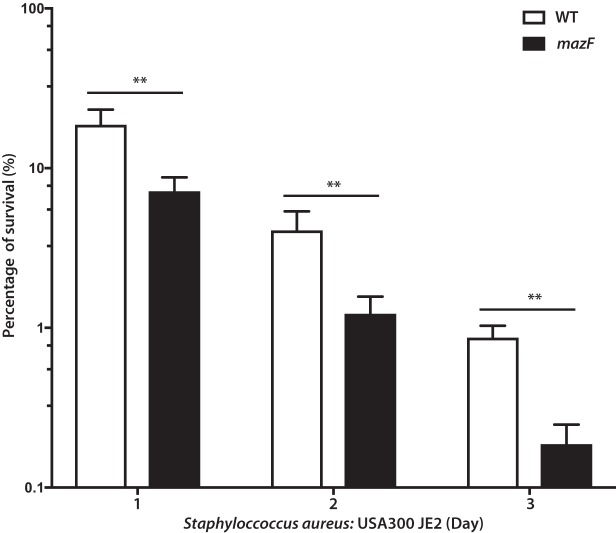
Loss of *mazF* expression decreases biofilm antibiotic tolerance in S. aureus. Mature S. aureus biofilm was cultured on surgical implant material (4 days on 12-mm titanium rods) and exposed to 10× MIC of cefazolin or vancomycin. Implants were then removed, sonicated, and plated to enumerate survivors on a daily basis over 3 days. Remaining biofilm on surgical implant material at each day was compared to the respective pretreated strain. All experiments were performed in triplicate. **, *P* < 0.01. Error bars represent 95% confidence intervals.

In Gram-negative bacteria, *mazEF* contributes to antibiotic tolerance and bacterial persisters (31–33). The roles of *mazEF* and other toxin-antitoxin systems in S. aureus antibiotic tolerance are conflicting and unclear (34, 35). We hypothesized that *mazEF* would contribute to biofilm antibiotic tolerance in S. aureus. Biofilm antibiotic tolerance was compared between the methicillin-resistant S. aureus (MRSA) strain JE2 and its corresponding strain disrupted for the *mazF* gene. Mature biofilm cultured on surgical implant material was exposed to 10× minimum inhibitory concentration (MIC) of vancomycin, and quantitative culture was used to assess remaining biofilm mass over 3 days. Loss of *mazF* expression had a statistically significant increased loss of biofilm mass compared to the wild-type control, demonstrating that loss of *mazF* expression decreased biofilm antibiotic tolerance (Fig. 3). These results were confirmed in two additional strains, Newman and SH1000, using both cefazolin and vancomycin (Fig. S2). For all three strains, there was no statistical difference in MICs between the wild-type and loss-of-function strains for cefazolin or vancomycin. Because JE2 is a MRSA strain, the sensitivity of cefazolin was not tested (see Table S1 in the supplemental material).

10.1128/mBio.01658-19.2 FIG S2 Loss of *mazF* decreases biofilm vancomycin and cefazolin tolerance in S. aureus. Mature biofilm grown on surgical implant material (4 days on titanium rods) was exposed to 10× MIC of cefazolin or vancomycin. Implants were then removed, sonicated, and plated to enumerate survivors on a daily basis over 3 days. A regression model was used to estimate the overall percentage of biofilm remaining at day 3 relative to pretreated biofilm. (A) Biofilms of Newman and SH1000 strains were exposed to cefazolin. JE2 was not included, as it is a MRSA. (B) Biofilm of JE2, Newman, and SH1000 strains were exposed to vancomycin. All experiments were performed in triplicate. *, *P* < 0.05; **, *P* < 0.01. Error bars represent 95% confidence intervals. Download FIG S2, EPS file, 1.5 MB. Copyright © 2019 Ma et al. 2019 Ma et al. This content is distributed under the terms of the Creative Commons Attribution 4.0 International license.

10.1128/mBio.01658-19.5 TABLE S1 Cefazolin and vancomycin MICs of nonbiofilm S. aureus. Download Table S1, DOCX file, 0.01 MB. Copyright © 2019 Ma et al. 2019 Ma et al. This content is distributed under the terms of the Creative Commons Attribution 4.0 International license.

### Lack of *mazF* expression altered planktonic antibiotic tolerance only when the doubling rate was altered.

**FIG 4:**
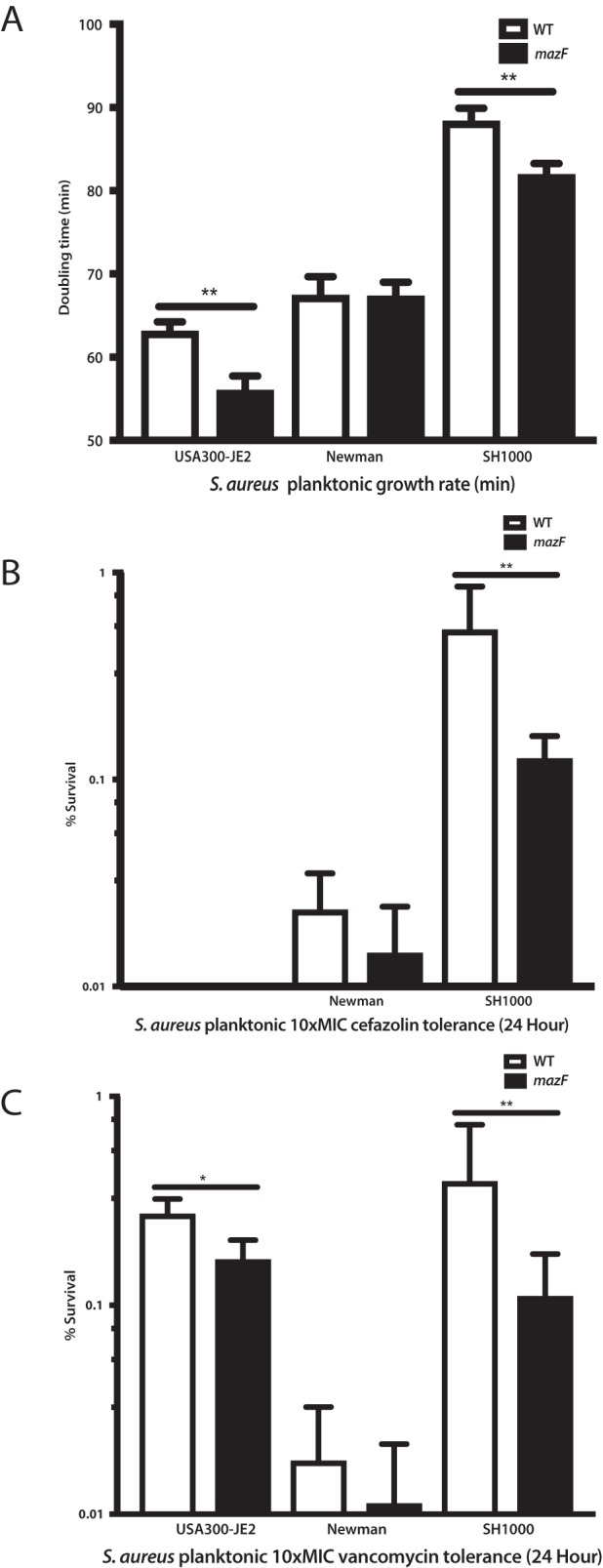
Loss of *mazF* expression increased planktonic growth and decreased vancomycin and cefazolin planktonic antibiotic tolerance in S. aureus. (A) Based on the cell growth curve, the doubling time of each strain was determined. Disruption of *mazF* from S. aureus resulted in a shorter doubling time in JE2 and SH1000 strains. (B) Disruption of *mazF* expression decreased the cefazolin planktonic antibiotic tolerance in strain SH1000. The JE2 strain was not included in cefazolin experiments, as it is methicillin resistant. (C) Disruption of *mazF* expression decreased the planktonic vancomycin tolerance in JE2 and SH1000 strains. All experiments were performed in triplicate. *, *P* < 0.05; **, *P* < 0.01. Error bars represent 95% CI (95% confidence intervals).

After observing these strong *mazF* biofilm phenotypes of increased biofilm formation and decreased antibiotic tolerance, we questioned whether a similar pattern would be observed in planktonic culture. The growth rates of these three S. aureus strains were compared after they exited stationary phase. Loss of *mazF* expression resulted in a statistically significant increased early logarithmic planktonic growth rate in S. aureus strains JE2 and SH1000, but this was not observed at each time point. When the early logarithmic doubling time was compared, only SH1000 and JE2 strains had a statistically increased doubling rate (Fig. 4A), while strain Newman did not. A similar pattern was observed with planktonic antibiotic tolerance; deletion or disruption of *mazF* decreased antibiotic tolerance only in the same strains that had an increase in doubling rate, JE2 and SH1000 (Fig. 4B and C).

### Disruption of *mazF* increased pathogenicity, limited the ability of S. aureus to transition from an acute to chronic infection, and inhibited antibiotic tolerance.

**FIG 5:**
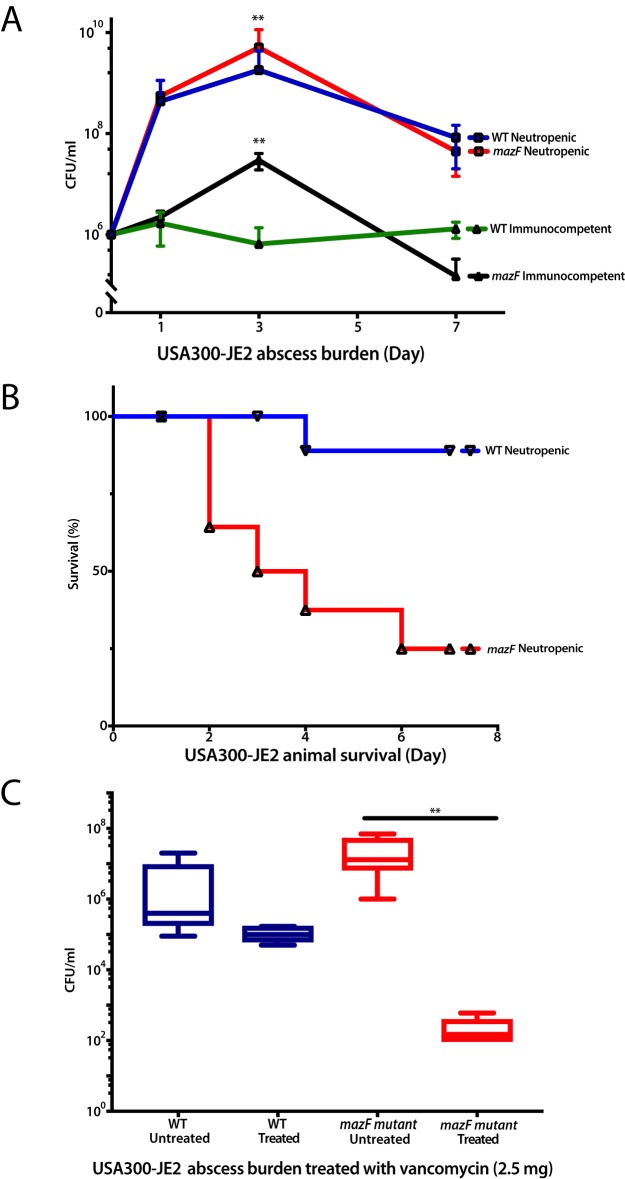
Loss of *mazF* expression increases pathogenicity and limits S. aureus ability to establish chronic infection. Bacterial abscess burden and animal survival were used to test the pathogenicity of wild-type S. aureus strain and its corresponding *mazF*::Tn strains. (A) In both neutropenic and immunocompetent groups, loss of *mazF* increases bacterial burden compared to wild-type strains, which was most apparent at 3 days postinfection (**, *P* < 0.01). (B) Mortality in neutropenic mice inoculated with strains that had no *mazF* expression was 25% on day 3 and 75% on day 7 postinfection. Mice inoculated with the wild-type strain had 0% mortality at day 3 and 10% mortality at day 7. (C) The strain that lost *mazF* expression was more sensitive to antibiotics than the wild-type control. After treatment with vancomycin, the loss of *mazF* expression had a 5-log-unit reduction in biofilm compared to the wild-type strain (**, *P* < 0.01).

If lack of *mazF* expression increased biofilm formation, we hypothesized that this increased proliferation would result in increased disease severity. To test this hypothesis, we used a murine abscess model. After inoculation in the hind limb, quantitative culture was used to determine abscess bacterial burden at increasing time points in the wild-type strain and the *mazF*::Tn strain. We selected the JE2 strain for these experiments, as it was the most clinically relevant strain. In immunocompetent mice, loss of *mazF* had a similar phenotype to *in vitro* observations with increased proliferation and biofilm mass compared to the wild-type strain. After 3 days of inoculation, the abscess burden decreased (Fig. 5A). To increase disease severity, we repeated experiments in neutropenic mice. Loss of *mazF* expression had increased proliferation and burden compared to wild-type bacteria at day 3. Further, we observed a more virulent and aggressive infection. Wild-type mice had almost 100% survival and transitioned to a chronic infection. Mice inoculated with the *mazF*-disrupted strain were unable to transition to a chronic infection, developed sepsis, and died with less than 25% survival by day 7 (Fig. 5B). Surprisingly, although a more aggressive infection was observed, the *mazF*-disrupted strain was more sensitive to antibiotics than the wild-type control. After inoculation, there was a larger decrease in bacterial burden after treatment with vancomycin in the *mazF*::Tn strain compared to the wild type (Fig. 5C). Together, these results supported the two *in vitro* phenotypes we observed and suggest that *mazF* contributes to a phenotype of decreased virulence and pathogenesis.

### Increased biofilm formation in a *mazF-*disrupted strain is *ica* dependent.

**FIG 6:**
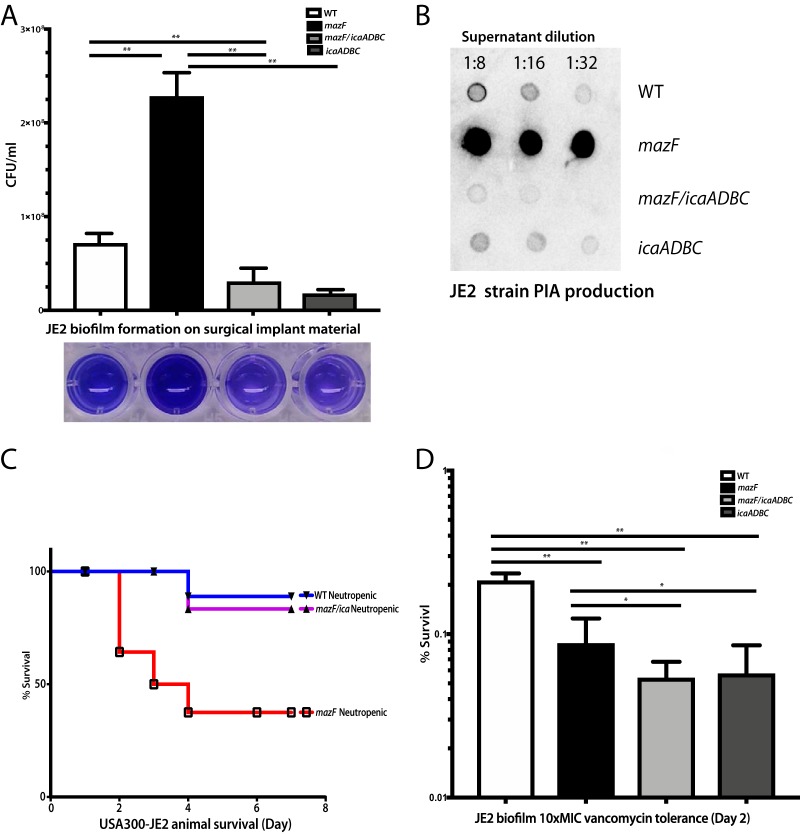
Increased biofilm formation in the *mazF* disruption strain is *ica* dependent. Quantitative CFU assay was used to measure the mass of biofilm. Biofilm formation in the *mazF*::Tn and *mazF*::Tn*/*Δ*icaADBC* strains were compared to the parental strain. (A) Biofilm formation of the *mazF*::Tn/Δ*icaADBC* strain was lower than that of strains lacking *mazF* expression alone. (B) PIA production in JE2 strains. The PIA production in the strain that lost *mazF* expression is much higher than that of other JE2 strains. (C) *mazF*::Tn/Δ*icaADBC* double loss-of-function strain reversed the animal mortality to wild-type strain levels. (D). Biofilm antibiotic tolerance of the double loss of function was lower than that of strains lacking *mazF* expression alone. All experiments were performed in triplicate. **, *P* < 0.01. Error bars represent 95% CI (95% confidence intervals).

After a phenotype for *mazF* and a possible role in pathogenesis were identified, we attempted to identify a mechanism behind its regulatory control. The intercellular adhesion gene cluster (*ica*) is composed of *icaA*, *icaD*, *icaB*, and *icaC* and encodes proteins that promote intercellular adhesion in many strains and species of *Staphylococcus* (36). Deletion of *mazF* in S. aureus results in increased biofilm formation that is *ica* dependent (37). To test the hypotheses that the phenotype of increased growth and pathogenesis from loss of *mazF* expression was *ica* dependent, we deleted the *icaADBC* genes from the *mazF*::Tn strain to generate a *mazF*::Tn/Δ*icaADBC* strain. This strain had lower biofilm formation than the wild-type and *mazF*::Tn strains as demonstrated by both a quantitative CFU assay on titanium rods and crystal violet assay. There was no difference in biofilm formation between the strains with disruption of *icaADBC* alone and disruption of both *mazF* and *icaADBC* (Fig. 6A). These observations in biofilm formation correlated with *icaADBC*-encoded polysaccharide intercellular adhesin (PIA) production. Disruption of *mazF* had a large increase in PIA production compared to the wild-type strain and in both strains when *mazF* and *icaADBC* were disrupted or when *icaADBC* alone was disrupted (Fig. 6B). This was further supported by quantifying expression levels of the *ica* transcripts (Fig. S3). Disruption of *mazF* resulted in higher expression levels of *icaA*, *icaB*, and *icaC* compared to the wild-type strain (Fig. S3). *icaD* expression levels were not analyzed, as the transcript length was small, preventing accurate measurement with quantitative reverse transcription-PCR (qRT-PCR). The neutropenic murine abscess model was repeated with the *mazF*::Tn/Δ*icaADBC* strain. The phenotype associated with loss of *mazF* expression was again suppressed. Mice inoculated with the *mazF*::Tn/Δ*icaADBC* strain had comparable survival to the wild-type control, whereas mice inoculated with the *mazF*::Tn strain had 50% survival (Fig. 6C). The *mazF*::Tn/Δ*icaADBC* strain overcorrected the *mazF*::Tn growth phenotype, confirming the roles of *icaA*, *icaB*, *icaC*, and *icaD* in *mazEF* function, which suggests that these four genes are likely involved in controlling other process outside the *mazEF* system. The ability of the *mazF*::Tn/Δ*icaADBC* strain to restore survival in the murine abscess model confirmed a role for *ica* in the control of biofilm formation in pathogenesis.

10.1128/mBio.01658-19.3 FIG S3 Loss of *mazF* expression increases *ica* expression in JE2 strain. Quantitative real-time RT-PCR analysis of *icaA*, *icaB* and *icaC* expression in JE2 strains. Δ*C_t_* values were used to quantify gene expression levels. The differences in the mRNA levels of *ica* genes for the wild-type JE2 and *mazF* loss-of-function strain are significant. All experiments were performed in triplicate. **, *P* < 0.01. Error bars represent 95% confidence intervals. Download FIG S3, EPS file, 0.7 MB. Copyright © 2019 Ma et al. 2019 Ma et al. This content is distributed under the terms of the Creative Commons Attribution 4.0 International license.

### Decreased biofilm antibiotic tolerance in a *mazF*-disrupted strain is not *ica* dependent.

We then tested the role of *icaADBC* in regulating *mazF* biofilm antibiotic tolerance. Mature biofilm was exposed to 10× MIC for vancomycin. *mazF*::Tn and *mazF*::Tn/Δ*icaADBC* strains had decreased vancomycin tolerance compared with the wild-type strain. Unlike the biofilm formation phenotype where deletion of *icaADBC* reversed the *mazF* disruption phenotype, loss of both *mazF* and *icaADBC* expression resulted in even less biofilm antibiotic tolerance than loss of *mazF* alone. This was supported by quantifying biofilm vancomycin tolerance in strains where only *icaADBC* expression was disrupted. Biofilm vancomycin tolerance was comparable between strains with loss of *icaADBC* expression and loss of both *mazF* and *icaADBC* expression. This demonstrated that *ica* genes were also involved in antibiotic tolerance (Fig. 6D).

### Loss of *mazF* expression did not alter *sigB* transcription.

The *sigB* operon is a master regulator in S. aureus that allows it to rapidly redirect transcriptional activities in response to stress (29). It has the potential to be a major regulator of S. aureus biofilm formation and virulence (38). The *sigB* operon is directly downstream from *mazEF*, and disruption of *mazF* expression could possibly alter *sigB* expression (29). We examined *sigB* expression and genes upstream and downstream of *mazF* to verify that neighboring gene expression was not altered. Under both planktonic and biofilm conditions, quantitative RT-PCR analysis demonstrated no change in expression of *sigB* and the *sigB*-dependent gene, *asp23* (alkaline shock protein 23), between each of the three strains with loss of *mazF* expression as compared to their respective wild-type control (Fig. S4A and B). We also examined the expression of the *rpoF*, *rsbW*, and *alr* genes which are directly upstream and downstream of *mazF*, based on genomic location and transcriptional order, using qRT-PCR. There was no statistically significant difference in *rpoF*, *rsbW*, and *alr* expression between these strains and their wild-type control (Fig. S4C). In these experiments, *mazF* expression remained low and beyond the limits of detection of our technique at a threshold cycle number (*C_t_*) greater than 35. These results demonstrate that loss of *mazF* did not alter expression of neighboring genes. The observed phenotype was related to the loss of *mazF* and not changes in the expression of *sigB* operon or other neighboring genes.

10.1128/mBio.01658-19.4 FIG S4 Loss of *mazF* expression had no effect on *sigB* operon expression. Quantitative real-time RT-PCR analysis of *sigB* (A) and *asp23* (B) expression in three S. aureus strains (JE2, Newman, and SH1000). Δ*C_t_* values were used to quantify gene expression levels. No significant differences in *sigB* or *asp23* expression were observed between the wild-type strain and strains that lost *mazF* expression in all three S. aureus strains. (C). Quantitative real-time RT-PCR analysis of the *sigB* operon with *rpoF*, *rsbW*, and *alr* transcripts. Δ*C_t_* values were used to indicate the expression levels of selected genes. Download FIG S4, EPS file, 1.5 MB. Copyright © 2019 Ma et al. 2019 Ma et al. This content is distributed under the terms of the Creative Commons Attribution 4.0 International license.

## DISCUSSION

The physiological roles of bacterial toxin-antitoxin systems remain unknown. In S. aureus, *mazEF* is a well-studied toxin-antitoxin system whose phenotype and physiological role remain elusive (32, 39, 40). Loss of *mazF* expression resulted in a phenotype of increased biofilm formation on surgical implant material and decreased biofilm antibiotic tolerance in all three S. aureus strains. In our murine abscess model, the phenotypes associated with *mazEF* contributed to a biofilm-dependent disease process that is consistent with most chronic bacterial infections and the clinical manifestation of surgical infections. Further mechanistic analysis supported a role for extracellular polysaccharide adhesins in the increased biofilm formation and pathogenesis when *mazF* expression is disrupted. Combined, these results suggest that *mazEF* helps regulate the transition between acute to chronic infection in S. aureus.

Regulation of growth and biofilm formation is a phenotype associated with TA systems. The mechanism of toxin-antitoxin systems includes an antitoxin that prevents the toxin from inducing growth arrest using a variety of tools (31, 41). After loss of *mazF* expression, we observed increased biofilm compared to its isogenic wild-type strain on fibrinogen-coated plastic and titanium *in vitro* and *in vivo* in our animal model. This supports the work of other groups where overexpression of *mazF* in S. aureus resulted in growth arrest (28) and *mazF* mutants had increased biofilm formation (37). This is the primary mechanism where bacteria are thought to become antibiotic tolerant from toxin-antitoxin systems; dormant bacteria are tolerant to an antibiotic whose main process is disrupting their metabolism.

There is evidence to suggest that TA systems play an important role in antibiotic tolerance based on multiple examples in Gram-negative species (33) as well as in acid-fast mycobacterium (24). Although there is less evidence for this phenotype in Gram-positive organisms, it has been suspected that a similar pattern exists in S. aureus. We observed a difference in biofilm antibiotic tolerance when *mazF* expression was disrupted compared to its wild-type strains (Fig. 3). We did not observe a difference in the MIC. This supports similar previous observations (37). Other groups have noted that in S. aureus, *mazF* transcription is altered by sub-MIC concentrations of tetracycline, penicillin, and linezolid (29). Despite generating greater biofilm formation, the loss of *mazF* expression demonstrated increased antibiotic susceptibility to clinically relevant antibiotics cefazolin and vancomycin. Combined, this provides strong evidence for a major role of *mazF* in S. aureus biofilm antibiotic tolerance.

We observed that the role of *mazF* in antibiotic tolerance appears to be correlated with growth. The phenotype of antibiotic tolerance was more weakly observed in planktonic S. aureus strains (Fig. 4). In planktonic culture, only loss-of-function strains with decreased doubling times compared to the wild-type strains were observed to have decreased planktonic antibiotic tolerance. Biofilm formation is thought to be associated with antibiotic tolerance. Surprisingly, we demonstrated that increased biofilm formation had the opposite effect and resulted in less antibiotic tolerance. This was likely the result of the bacteria having a higher rate of metabolic activity compared to the normal decreased metabolic state of the biofilm. This supports other results suggesting that antibiotic tolerance and persister formation is based on ATP levels. Multiple mechanisms probably exist to support persister cell formation and antibiotic tolerance, including the stringent response (35).

Biofilm formation is an important step for S. aureus to establish an infection. This is regulated by polysaccharide intercellular adhesin (PIA/PNAG [poly-*N*-acetylglucosamine]) encoded by the *ica* operon (42). On the basis of this and our observation that loss of *mazF* expression increased biofilm formation and PIA production, we speculated that *mazF* inhibits biofilm formation by decreasing *ica* transcription. A *mazF*::Tn/Δ*icaADBC* strain reversed the *in vitro* and *in vivo* phenotypes from loss of *mazF* expression (Fig. 6). This *mazF*::Tn/Δ*icaADBC* strain had pathogenicity similar to that of the wild-type strain. This provides evidence that *ica*-mediated biofilm formation and pathogenicity are inhibited by *mazF*. This supports other groups’ observations that S. aureus biofilm formation is dependent on *mazF* mRNA interferase activity (37).

S. aureus infections are typically acute. Although there is a range of pathogenesis from simple, superficial abscesses to life-threatening systemic sepsis, the outcomes of these disease processes resolve over a limited time period. An exception is surgical infection where chronic infections can develop over an extended period of time and biofilm formation plays an important physiological role (5, 11). Regulation of growth, biofilm formation, and antibiotic tolerance could have important roles of bacterial physiology in this disease state. S. aureus biofilm formation is an essential step in establishing infection and pathogenicity (43, 44). Surprisingly, although loss of *mazF* created a more virulent organism with higher lethality, these infections were also more susceptible to antibiotics. Combined, these results suggest that *mazF* expression inhibits biofilm formation and increases antibiotic tolerance, allowing the bacteria to transition to a chronic infection that is more challenging to treat. A simplistic and binary definition of the transition between an acute to chronic infection is mortality. In our studies, loss of *mazF* expression resulted in increased virulence and infection severity. This prevented a transition to a chronic infection and, instead, resulted in death. This demonstrates a physiological role for toxin-antitoxin systems during infections. MazEF toxin-antitoxin systems not only make the bacteria more tolerant to antibiotics but make the bacteria more tolerant to the host.

## MATERIALS AND METHODS

### Bacterial strains, plasmids and growth conditions.

Staphylococcus aureus strains SH1000, Newman, and JE2 are in clonal complex 8. SH1000 is a *rsbU*^+^ derivative of NCTC 8325, which encodes a positive regulator of *sigB* activity. It was initially derived from a corneal ulcer and is a primary strain used for genetic manipulation (45). Newman was a pathogen isolated from a newborn infection in the 1950s (46). JE2 is a USA300 MRSA strain isolated from an inmate in the Los Angeles County Jail in California, USA (47). USA300 JE2 was selected as the primary strain to be tested in our *in vivo* model for three reasons. First, it is the most clinically relevant. Second, USA300 clones have the highest growth rate compared to other common S. aureus strains (48) which was a primary phenotype we were interested in exploring. Finally, the largest difference in phenotypes observed during our *in vitro* experiments occurred in this strain. All bacterial strains and plasmids used in this study are listed in Table 1. S. aureus strains were cultured in Trypticase soy broth (TSB) medium with or without antibiotics.

**TABLE 1:** Bacterial strains and plasmids used in this study

Strain or plasmid	Relevant genotype and/or characteristic(s)	Source and/or reference
Strains		
RN4220	Heavily mutagenized NCTC 8325-4	A. L. Cheung (49)
Newman-WT	Overexpresses *clfA* and *sae*	A. L. Cheung (29)
Newman-Δ*mazEF*	Newman Δ*mazEF*	A. L. Cheung (29)
SH1000-WT	Derived from NCTC 8325	B. Löffler (30)
SH1000-Δ*mazF*	SH1000 Δ*mazF*	A. L. Cheung (29)
JE2-WT	FPR3757 *pvl* positive	ATCC
JE2-*mazF*::Tn	NE1833 with JE2 *mazF*::Tn	Nebraska Tn Mutant Library
JE2-WT-Spec	WT JE2 with empty spectinomycin vector	This study
JE2-comp	JE2 *mazF* complement	This study
JE2-*mazF*::Tn*/*Δ*ica*	JE2 *mazF*::Tn/Δ*icaADBC*	This study
*E. coli* DH10B	General purpose competent cells for cloning	Thermo Fisher Scientific

Plasmids		
pKFT	5.7-kb temperature-sensitive shuttle vector; Amp^r^ Tet^r^ in *E. coli*, Tet^r^ in *S. aureus*	M. Inouye (37)
pLZ12-Spec	Shuttle vector with pWV01 origin; Spec^r^ in *E. coli* and *S. aureus*	50
pFK74	pKFT containing regions upstream and downstream of the *icaADBC* genes	37

### Genomic bacterial DNA isolation.

Genomic DNA was isolated from S. aureus samples by following manufacturer’s instructions (MasterPure Gram-positive DNA purification kit; Lucigen, USA). Briefly, a single colony from a TSB plate was inoculated in TSB medium and grown overnight at 37°C in an orbital shaker. The culture (1.5 ml) was centrifuged, and the pellet was resuspended in 150 μl Tris-EDTA (TE) buffer. The bacteria were placed in lysis buffer at 37°C until the bacterial cell wall was destroyed and treated with proteinase K. After protein precipitation reagent was added, the pellet was centrifuged at 4°C for 10 min at 12,000 × *g*. The pellet was rinsed with 70% ethanol and resuspended in TE buffer.

### Isolation of RNA and quantitative RT-PCR analysis.

RNA isolation and quantitative reverse transcription-polymerase chain reaction (qRT-PCR) were performed by following the manufacturer’s instructions. Briefly, S. aureus was grown in 4 ml of TSB medium at 37°C for 16 h or grown on titanium rods for 72 h. Bacteria were collected by centrifugation, and the pellet was resuspended in TE buffer by sonication and vortexing. Lysostaphin (500 μg/ml) (Sigma-Aldrich) was added to the resuspended bacteria and incubated at 37°C for 15 min. Total RNA was extracted using TRIzol Max bacterial RNA isolation kit (Thermo Fisher Scientific). Single-stranded cDNA was created from reverse transcription of the RNA using SuperScript IV reverse transcriptase (Thermo Fisher Scientific). The newly synthesized cDNA was used immediately or frozen at −80°C.

Quantitative RT-PCR analysis was performed using the CFX384 real-time system (Bio-Rad, Richmond, CA) and PowerUp SYBR green master mix (Thermo Fisher Scientific). The cycling conditions were 50°C for 10 min and 95°C for 5 min, followed by 45 cycles, with 1 cycle consisting of 95°C for 10 s and 60°C for 30 s. For all samples, the threshold cycle number (*C_t_*) at which the fluorescence values became logarithmic was determined. The Δ*C_t_* value was calculated for each sample as the difference between the sample *C_t_* and the control *C_t_*. The *C_t_* value of *mazF* in the *mazF* deletion strain was greater than 36. We did not calculate a Δ*C_t_* value if the *C_t_* value was more than 35, as this was beyond the limit of accurate measurement using SYBR green in quantitative real-time PCR.

### Creation of the *mazF* complementary strain.

The complete *mazF* gene was amplified from 860 bp upstream of the *mazF* open reading frame, including the promoter region, in strain JE2 by PCR and cloned into a pLZ12-spec shuttle vector. The transformed *mazF* expression vector was transformed into the *mazF*::Tn strain and selected with 200 μg/ml spectinomycin.

### Creation of the *icaADBC* gene deletion using pKFT vector.

A JE2 double *mazF*::Tn/Δ*icaADBC* strain was created from the base JE2 *mazF*::Tn strain using a previously described protocol (51). Briefly, the allelic replacement vector pFK74 containing regions upstream 1.1 kb and downstream 0.9 kb of the *icaADBC* gene was first transformed into DNA restriction system-deficient S. aureus RN4220, and then a modified plasmid was isolated and electroporated into *mazF*::Tn strain (NE1833) from the NARSA NR-48501 library. Transformants were selected at 30°C on TSB plates containing tetracycline. A single colony transformant was cultured at 30°C in TSB medium containing tetracycline in an orbital shaker. Integration of the plasmid into the chromosome by a single crossover event was achieved by incubation at 42°C on TSB plates containing tetracycline. Correct homologous recombination of the target region was verified by PCR using primer set of pUC-UV (5′-CGACGTTGTAAAACGACGGCCAGT-3′, plasmid) and *icaADBC* 5′-up (5’-CCATCACATAGGCGCTTATCAA-3′, chromosome) or pUC-RV (5′-CATGGTCATAGCTGTTTCCTGTG-3′, plasmid) and *icaADBC* 3′-dn (5′-GAAGCAACGCACAAAGCATTA-3′, chromosome). Integrants were grown at 25°C overnight with shaking in 10 ml TSB without any antibiotics. Bacteria were serially diluted and plated on TSB plates at 42°C. The excision of the plasmid region in the chromosome by a second crossover event was screened for by isolation of tetracycline-sensitive colonies by replica plating candidates on TSB plates versus TSB plates containing tetracycline (3 μg/ml). Integrants were cultured overnight at 37°C. Then, the markerless deletion mutants were screened by PCR using primers *icaADBC*-1 (5′-AAAAAGATCTTTAGTAGCGAATACACTTC-3′) and *icaADBC*-4 (5′-TACAAGATCTTTGGCATCATTTAGCAGAC-3′) from tetracycline-sensitive colonies. The strain with *icaADBC* deletion was screened by PCR and confirmed by DNA sequencing.

### Cell growth curve and doubling time.

Approximately 1 × 10^6^ cells were added from overnight culture to fresh TSB medium containing 0.25% glucose and incubated at 37°C. The optical density at 600 nm (OD_600_) absorbance was measured every h during a 24-h period (Infinite M200; Tecan). Calculation of the doubling time was based on these measurements.

### Biofilm formation assay.

Four titanium rods (12 mm) per well were incubated in TSB growth medium inoculated with 1 × 10^4^ CFU S. aureus for 24 to 96 h. Titanium rods were then washed three times with 1 ml of phosphate-buffered saline (PBS) and then sonicated for 30 min in 1 ml fresh TSB medium. After serial 1:10 dilution, the bacterial concentration (CFU/milliliter) was determined via colony-forming unit (CFU) assay on TSA II blood agar plates (Thermo Fisher Scientific, USA). A semiquantitative adherence assay was performed on 96-well tissue culture polystyrene plates (Sigma-Aldrich, USA). The wells on the plates were coated with 200 μl of PBS containing 5 μg/ml fibrinogen (Sigma-Aldrich, USA) overnight at 4°C. The wells on the plates were washed three times with PBS and then blocked with 100 μl of a 2% bovine serum albumin (BSA) solution for 1 h at 37°C. The wells were carefully washed three times with 100 μl of PBS; 100 μl of bacteria (approximately 1 × 10^7^ cells) was added to the appropriate wells and incubated for 24 h at 37°C. The wells were washed four times with 100 μl of PBS. Bacteria were fixed with 100 μl of 10% formaldehyde (Sigma-Aldrich, USA) for 10 min. Then 100 μl of 0.2% crystal violet (Sigma-Aldrich, USA) was added to each well for 10 min, and cells were washed four times with distilled water. Wells were dried in air for 2 h and then 100 μl of 30% acetic acid (Fisher Scientific, USA) was added to dissolve the crystal violet. The absorbance was measured at 590 nm (Infinite M200; Tecan).

### MIC assay.

A single colony from an overnight agar plate was inoculated in 5 ml TSB medium to achieve the specified inoculum turbidity by comparing to a 0.5× McFarland turbidity standard (∼1 × 10^8^ CFU/ml). A sterile swab was placed in the inoculum suspension and streaked across the entire agar surface six times, rotating the plate to evenly distribute the inoculum. An Etest MIC test strip (Liofilchem, Italy) was applied with sterile forceps. Agar plates were then incubated in an inverted position at 37°C overnight.

### Biofilm and planktonic antibiotic tolerance assay.

For the biofilm assay, S. aureus strains were cultured on surgical implant material (12-mm titanium rods) for 4 days with a daily medium exchange to form mature biofilm. The biofilm was then exposed to 10× MIC of cefazolin or vancomycin for 3 days with daily medium exchange. Implants were then washed, removed, sonicated, and plated to enumerate survivors (CFU assay) each day. For the planktonic assay, S. aureus strains were cultured in fresh TSB medium overnight. The next day, 1:100 diluted overnight culture was added to fresh TSB medium and grown to around 0.5× McFarland turbidity under 37°C. The bacteria were exposed to 10× MIC of cefazolin or vancomycin for 4 h and 24 h and plated to enumerate survivors (CFU assay). The percentage of survival was calculated at each time point compared to the initial time point of untreated cultures. All experiments were performed in triplicate.

### PIA production was detected by dot blotting assay.

The polysaccharide intercellular adhesin (PIA) production quantification protocol has been described previously (52). In brief, S. aureus was grown in TSB containing 0.25% glucose overnight, and 1 ml of each culture was harvested and resuspended in 50 μl of 0.5 M EDTA (pH 8.0). Cells were incubated for 5 min at 100°C and centrifuged to pellet the cells. The supernatant was treated with 10 μl of proteinase K (20 mg/ml; Sigma-Aldrich) for 30 min at 37°C. The supernatant was diluted with Tris-buffered saline (20 mM Tris-HCl, 150 mM NaCl [pH 7.4]), spotted on a nitrocellulose membrane, dried, blocked with 3% BSA (Sigma-Aldrich) in PBS, and incubated at room temperature with wheat germ agglutinin-horseradish peroxidase (WGA-HRP)-labeled lectin for 2 h at a dilution of 1:1,000 (catalog no. L3892; Sigma). The membrane was then washed three times with Tris-buffered saline with Tween 20 (TBST) buffer. The membrane was rinsed with water, and images of enhanced chemiluminescence (ECL) were collected by ChemiDoc Touch Imaging System (Bia-Rad).

### Mice, neutropenic thigh model, and S. aureus strain administration.

We selected the JE2 strain for *in vivo* experiments as the growth phenotype was most pronounced. Eight-week-old B57BL/6J mice were purchased from the Jackson Laboratory (Bar Harbor, ME, USA). All animal protocols used for these experiments were approved by the University of Pittsburgh’s Institutional Animal Care and Use Committee. Mice were rendered neutropenic by two 100-μl intraperitoneal injections of cyclophosphamide (150 mg/kg of body weight 3 days preinfection and 100 mg/kg 1 day preinfection). Mice were anesthetized by 2% isoflurane, hair was removed from the leg, and the leg was treated with betadine. An inoculation volume of 100 μl, 1 × 10^6^ CFU of JE2-WT, JE2-*mazF*::Tn strain, or JE2-*mazF*::Tn strain/*ΔicaADBC* was injected into the thigh. Mice were monitored for weight loss, leg swelling, ambulatory abilities, signs of sepsis, and death. Mice were sacrificed at 1, 3, and 7 days postinfection. A ∼5 × 5 mm piece of thigh muscle from infection site was obtained and placed in 1% Tween 20 in PBS on ice. Abscess samples were sonicated 10 min, and colony-forming unit (CFU) assay was performed on blood agar plates to quantify bacterial burden.

### Statistical analysis.

Statistical analysis was based on the number of populations and comparisons. Student *t* test was used for two populations. One-way analysis of variance (ANOVA) and two-way ANOVA was used for comparing multiple populations across either one condition or two conditions, respectively. To determine antibiotic tolerance, multilevel mixed-effects linear regression models were constructed to compare the rate of change in CFU/milliliter over time between the wild type and strains with loss of *mazF* expression. The outcome, CFU/milliliter, was natural log transformed to produce approximately normally distributed values before fitting models via maximum likelihood estimation (MLE). Bacterial type (wild type [WT], Δ*mazF*), time, and a type-by-time interaction were included as fixed effects in the multilevel models, and the baseline concentration was accounted for. Random effects for experiment, as well as group (nested within experiment), were included in all models to adjust for within-cluster correlation. Of primary interest were the type-by-time interaction coefficients, which reflect the degree to which the rate of decline in log CFU differs between wild-type and Δ*mazF* bacteria. After fitting the models, the estimated interaction coefficients were back-transformed to provide interpretable results on the original, nonlogarithmic CFU/milliliter scale.
