# Supplementary material for: The Toxin-Antitoxin MazEF Drives Staphylococcus aureus Biofilm Formation, Antibiotic Tolerance, and Chronic Infection
Source: mBio. 2019 Nov 26;10(6):e01658-19. doi: 10.1128/mBio.01658-19 (PMC6879715; doi:10.1128/mBio.01658-19)
Supplement: TABLE S1 [file mBio.01658-19-st001.docx]

Supplemental Table 1 Cefazolin and vancomycin MICs of non-biofilm *S. aureus*

| Strains | MICs of cefazolin  (means±SD) (µg/ml) | MICs of vancomycin  (means±SD) (µg/ml) |
| --- | --- | --- |
| Newmen-WT | 0.29±0.08 | 2.33±0.58 |
| Newmen-Δ*mazEF* | 0.34±0.08 | 2.67±0.58 |
| SH1000-WT | 0.12±0.02 | 1.67±0.29 |
| SH1000-Δ*mazF* | 0.12±0.00 | 1.83±0.29 |
| JE2-WT | ND | 1.08±0.38 |
| JE2-*mazF::*tn | ND | 0.92±0.14 |

The MIC difference between the *mazF* loss of function and wild type strains is not significant (p>0.25). ND indicates not determined as JE2 is a methicillin resistant strain of *S. aureus*.
